# Factors associated with cardiovascular disease: A comparative study of the UK Asian diaspora and residents of India

**DOI:** 10.1371/journal.pone.0301889

**Published:** 2024-04-16

**Authors:** Mubarak Patel, Olalekan Uthman

**Affiliations:** 1 Warwick Evidence, Warwick Medical School, University of Warwick, Coventry, United Kingdom; 2 Warwick Medical School, University of Warwick, Coventry, United Kingdom; University of Georgia, UNITED STATES

## Abstract

**Introduction:**

The aim of this paper is to investigate what factors are associated to cardiovascular disease and what differences exists between Asians living in the UK (from the English Longitudinal Study of Ageing) and the Asians living in India (from the Longitudinal Ageing Study in India).

**Methods:**

Logistic regression was used to investigate how demographic and physical performance factors were associated with cardiovascular disease using data from Wave 6 of the English Longitudinal Study of Ageing and Wave 1 of the Longitudinal Study of Ageing in India, with the main variable of interest being country of residence, Asians in England or Asians in India.

**Results:**

A total of 83,997 participants were included in the analyses. In the primary analysis, 73,396 participants from LASI were compared to 171 Asians in ELSA. After adjusting for age, blood pressure, resting heart rate, sex, waist circumference, gait, handgrip strength and standing balance, there was a statistically significant difference for the outcome of CVD between Whites ELSA (reference) and the participants of LASI (odds ratio = 0.77; 95% confidence interval = 0.60 to 0.99). There were no significant differences in CVD between the LASI participants, Asian ELSA, and the Non-White but not Asian ELSA groups.

**Discussion:**

No difference was found between Asians that live in India compared to ethnic minorities living in England, including Asians, after adjusting for confounders, but was found between Whites in ELSA compared to LASI participants. A key limitation was the massive disparity in sample sizes between the ELSA subgroups and LASI. Further work is required where comparable sample sizes and longitudinal analyses allow trends to be identified and to investigate the factors associated with the difference in CVD between two similar ethnicities living in distinct locations.

**Conclusion:**

After adjusting for risk factors, there was no difference in CVD between localised Asians and the ethnic minorities in the UK, but there was a difference between the majority ethnicities in the respective countries.

## 1. Introduction

Cardiovascular diseases (CVD) are a group of diseases affecting the heart and blood vessels and is the leading cause of death worldwide [1]. In 2017 alone, CVD caused almost 18 million deaths worldwide, equivalent to 330 million years of lost life and 36 million years living in disability [2,3]. Between 1990 and 2019, CVD incident cases increased by 77% to 55.45 million and deaths rose almost 54% to 18.56 million [4]. This surge in cases and deaths underscores the need for comprehensive strategies to address the growing impact of CVD on global public health.

In 2012, CVD was the most common cause of death in the United Kingdom (UK) for females, and the second most for males [5]. As of 2022, one in eight men and one in fourteen women die from coronary heart disease (CHD) in the UK. CHD is responsible for more than twice as many deaths in UK women compared to breast cancer (the most commonly diagnosed cancer in women), similarly for premature deaths (women under 75 years of age). Moreover, an estimated 7.6 million people in the UK are living with CVD [6] which is expected to increase despite CVD-related mortality having been steadily decreasing in the UK, where it has done so since the 1960s [7].

A similar story has unfolded in the developing world since the turn of the century, where almost 80% of global CVD cases occur [8], and patients in low and middle-income countries had higher rates of major CVD and death compared to than in high-income countries [9]. However, there remains a glaring disparity in research output and disease burden between lower and higher-income countries [10]. CVD has become the leading cause of mortality in India [11], amounting to over a quarter of all mortality [12] with predictions of an estimated 3,070 years of life lost due to CVD per 100,000 people in India by 2030 [13]. CVD risk factors such as prevalence of overweight people, alcohol use, raised blood pressure, among others, have all seen increases over 20 years [14] in lower-income countries due to socioeconomic shifts being more in line with Western values and habits [15]. Furthermore, diseases such as coronary heart disease are affecting Indians at least 5–6 years earlier than their Western counterparts [16].

Most cases of CVD and CVD-related mortality can be attributed to a small number of common, modifiable risk factors, including grip strength and physical activity [17,18]. Regular exercise is considered to be a critically underused preventive strategy that can prevent CVD in older adults [19], even low doses of exercises such as a 30-minute brisk walk at least four times a week is associated with improved CVD outcomes [20]. A systematic review of healthy adults with no existing CVD concluded that increased physical activity was associated with a reduced risk of CVD [21]. In older adults, higher levels of physical activity is associated with lower risk of CVD [22,23] and, generally, higher fitness is associated with improved survival [24].

One study investigated coronary risk factors in people from the Indian subcontinent living in London, United Kingdom (UK), and their siblings living in Punjab, India [25], and found that those living in London exhibited greater levels of coronary risk factors than their siblings living in India.

A general slight decrease in age-related CVD mortality rate in developed countries, compared to an increased mortality rate in developing countries, whilst people from the UK demonstrating worse cardiovascular risk factors compared to India, begs the question of how these populations differ.

The primary objective of this paper was to compare cardiovascular events of the ageing Asian and British Asian (henceforth ELSA A+BA) population living the UK to the ageing population living in India, to investigate how physical performance and other risk factors are associated to CVD, and the differences that exist between the two populations. These groups were chosen as the UK is a key country of interest and due to the readily-available data of Indian Asians using two sister-studies which allowed direct comparability between the two groups. The secondary aim was to compare other ethnic minorities and the white population of the UK to the population of India. This addition allowed the exploration of potential variations in CVD risk across diverse ethnic groups within the UK and provide comprehensive insights that contribute to the understanding of health disparities.

## 2. Material and methods

### 2.1. Data sources

#### 2.1.1. English longitudinal study of ageing

Data for participants in England were drawn from the English Longitudinal Study of Ageing (ELSA) [26]. It is a representative sample of people aged 50 years and over, living in private households in England. The original sample was selected from participants who previously responded to the Health Survey for England (HSE) between 1998 and 2001. Participants are interviewed every two-years, known as ‘waves’ to measure changes in health, economic and social circumstances, and the sample has been, thus far, refreshed in Waves 3, 4, 6, 7, and 9 so that the study remains representative to the UK older 50 population. All participants of ELSA gave written informed consent and this study has been approved by the NRES Committee South Central—Berkshire on 28th November 2012 (11/SC/0374).

#### 2.1.2. Longitudinal ageing study in India

Data for participants from India were drawn from the Longitudinal Ageing Study in India (LASI) [27]. Launched in 2016, LASI is a nationally representative biennial panel survey of 73,396 older adults aged 45 years and above across all 28 states and eight union territories of India. Wave 1 of LASI collected data between April 2017 to December 2018 (collection for the state Sikkim was carried out in 2020–2021) and provides in-depth data on ageing, economics, health status, social relationships and support, family, and life satisfaction. Importantly, LASI is internationally harmonised with the Health and Retirement Study (HRS) and its sister studies globally to enable cross-national comparison, which enables comparability with the ELSA project. All participants of LASI gave written informed consent and this study has been approved by the Indian Council of Medical Research (ICMR) extended the necessary guidance and ethical approval for conducting the LASI.

#### 2.1.3. Choosing comparable waves

As of 2022, there are 9 waves of data for ELSA and only one wave of data for LASI. Data collection for Wave 1 of LASI took place between April 2017 and December 2018. The corresponding Wave of ELSA is either Wave 8 (data collection between 2016 to 2017), or Wave 9 (data collection between 2018 to 2019). However, only the even-numbered Waves collected data related to physical examination and performance data, so Wave 9 was ruled out. In addition, data on standing balance score was not collected at Wave 8, but was collected at Wave 6 (data collection between 2012 to 2013). Therefore, data from Wave 1 of LASI was directly compared to data from Wave 6 of ELSA.

#### 2.1.4. Ethical approval

ELSA Wave 6 received ethical approval from the NRES Committee South Central—Berkshire on 28th November 2012 (11/SC/0374).The Indian Council of Medical Research (ICMR) extended the necessary guidance and ethical approval for conducting the LASI.

### 2.2. Outcome variable

In ELSA wave 6, participants were not explicitly asked if they ever diagnosed with CVD. Instead, they were asked if they were diagnosed with either angina, myocardial infarction, congestive heart failure, or stroke. These were combined to create an overall binary CVD variable.

In LASI wave 1, participants were asked if they were ever diagnosed with chronic heart disease. The responses were then broken down into individual CVD: rheumatic heart disease, congenital disorders, arrhythmia, congestive heart failure, coronary disease, stroke, or other. The responses to the first question was used as the outcome for LASI.

### 2.3. Exploratory variables

The main variable of interest was ethnicity, comparing the Asian population of LASI (henceforth Asian LASI) to the following:

- Asian or British Asian population ELSA (henceforth Asian ELSA).
- Non-White population from ELSA who were not Asian (henceforth Non-White but not Asian ELSA).
- White population from ELSA (henceforth White ELSA).

Other baseline characteristics were chosen based on known factors that are associated with CVD [28–30], but also factors that were measured in both LASI Wave 1 and ELSA Wave 6. Some variables associated with CVD, such as smoking, were recorded at specific waves only and thus were unavailable at the waves analysed in this study. Age was measured in years. Body mass index (BMI) was calculated using each participant’s height and weight, and then grouped according to World Health Organisation (WHO) guidelines [31] into normal, underweight, overweight, and obese categories. Systolic (SBP) and diastolic blood pressure (DBP) was measured in mmHG. Highest level of education attained was grouped into none attained, foreign/other, below secondary, secondary or equivalent, advanced-level (A-level; post-secondary) or equivalent, and degree or above. Employment was grouped into employed or not employed. Heart rate was measured in beats per minute (BPM). Marital status was grouped into married or not married. Gender was split into males or females. Waist circumference was measured in cm.

Physical performance has a known association to CVD severity and was measured using a variety of tests. Grip strength, both dominant and non-dominant hand, was measured in kilograms. LASI averaged two separate grip strength readings and ELSA averaged three measurements.

The usual gait speed test measured how quickly a participant can walk a 4-meter course at their usual speed. Scoring is then categorised as follows:

- 0 points if unable to do the walk,
- 1 point if time is more than 8.70 seconds,
- 2 points if time is between 6.21 to 8.70 seconds,
- 3 points if time is between 4.82 to 6.20 seconds,
- 4 points if time is less than 4.82 seconds.

Standing balance was scored by combining participants’ performance in three different tests: side-by-side stand, semi-tandem stand, and tandem stand. Performance in these three tests are scored and then summed, giving a maximum total of 4 points if a participants completes all three balance tests for 10 seconds each, and a minimum score of 0 if a participant fails or does not attempt all three tests.

All baseline and performance variables were measured during Wave 1 for LASI participants, and Wave 6 for ELSA participants.

### 2.4. Statistical analysis

Categorical variables were summarised as number and percentage. Continuous variables were tested for normality using quintile-quintile and normal probability plots. In both plots, the data is normally distributed if the points on the graph form an approximate straight line. Continuous variables that were normally distributed were summarised as mean and standard deviation, and those that were not normally distributed were summarised as median and inter-quartile range.

As the outcome variable was binary, a logistic regression model was used to model CVD with the main variable of interest, ethnicity, and the aforementioned baseline and performance variables. First, each variable was modelled alone in a univariate model with CVD as the outcome to assess their unadjusted association with CVD. Those whose p-value ≤ 0.10 were then modelled in the multivariate model. A significance level of 10% was used in this step to ensure that factors with even a borderline-significant association was included in the final model before the model was finalised.

Assumptions of the binary logistic regression modelling process were tested in the model post-estimation. The following were the tests used.

Specification error is based on the assumption that the logit of the outcome is a linear combination of the independent variables, and was tested using the ‘linktest’ command in Stata, which uses the linear predictive value ($\hat{h}$) and linear predicted squared value (${\hat{h}}^{2}$). In the output of this command, $\hat{h}$ is statistically significant then meaningful predictors have been chosen for the model. If ${\hat{h}}^{2}$ is statistically significant, then either relevant variables were omitted or the link function is incorrectly specified. In this case, as there is a binary outcome, the correct link function is likely to be logit, so a statistically significant ${\hat{h}}^{2}$ would indicate that relevant variables were omitted.

Goodness of fit was tested using the Hosmer and Lemeshow’s goodness-of-fit test [32]. This test investigates how closely the predicted frequency matches the observed frequency. A good fit would yield a large p-value.

Multicollinearity occurs when two or more independent variables in the model are approximately determined by a linear combination of other independent variables in the model. Stata automatically removes variables that are perfect linear combinations of others. To measure multicollinearity, variance inflation factor (VIF) and tolerance were calculated for continuous variables. VIF is an indicator of much the inflation of the standard error of a variable could be caused by collinearity, and tolerance is an indicator of how much collinearity a linear regression analysis can tolerate. If variables are orthogonal (unrelated) to each other, then both VIF and tolerance should be close to one. Weight and height were not modelled due to the inclusion of BMI.

Influential observations may skew the regression estimates, therefore any strongly influential outliers were removed from any sensitivity analyses. These were identified using studentised Pearson residuals, deviance residuals, and leverage statistic–the hat diagonal/value. Observations whose Pearson residuals or deviance residuals >|2|, or whose hat value >2* average leverage, were removed.

Usual gait speed and standing balance forms part of the Short Physical Performance Battery (SPPB) test which is a measure of frailty [33]. The other test which is part of the SPPB is the repeated chair stand, where a participant attempts to rise from a chair five times without using their arms in the fastest time. The repeated chair stands test was performed in ELSA but not in LASI, thus the gait speed and standing balance tests were analysed separately, and were not combined in the analyses. All continuous variables were centred, either on their respective grand means (rounded to zero decimal points) when taking into account both the LASI and ELSA populations together, or based on published averages. Age was centred on 50 years as this was the minimum age of ELSA participants. Heart rate was centred on 60 bpm as this is average heart rate. Centring of variables was defined as shown in Table 1.

**Table 1:** Values that continuous variables were centred on for primary and secondary analyses.

Variable	Value centred on	Description of centred value
Age	50	Minimum age of ELSA participants
Diastolic blood pressure	81	Grand mean*
Heart rate	60	Average beats per minute[34]
Systolic blood pressure	129	Grand mean*
Waist circumference	87	Grand mean*
Grip strength (dominant hand)	22	Grand median*
Grip strength (non-dominant hand)	20	Grand median*

* Grand mean and grand median is based on the overall mean and median from participants of ELSA and LASI combined.

Abbreviations: ELSA = English Longitudinal Study of Ageing.

The following sensitivity analyses were undertaken involving the estimation of the Indian population within the ELSA dataset using bootstrapping with replacement techniques. First, based on the 2011 census of England and Wales, it was determined that approximately one-third of individuals identified as Asian British had Indian ancestry [35]. Consequently, one-third of the Asian or Asian British cohort within ELSA underwent 1,000 bootstrapping iterations and was subsequently compared to LASI. Second, bootstrapping was used to make the sample sizes of the ethnic groups of ELSA equal to the full sample size of LASI, the largest group analysed. Thirdly, a similar approach to make the group sizes of the larger groups to be equal to the sample size of the Asian or Asian British cohort of ELSA, the smallest group analysed. Missing data was excluded from analysis. Multiple imputation was then employed as a sensitivity analysis and compared to the primary models where there were no changes to the direction of effect and statistical significance.

All analysis was performed using StataSE 17 (64-bit) [36] and statistical significance was set to 5%.

## 3. Results

### 3.1. Baseline characteristics

There were a total of 83,997 participants in the analysis. LASI participants amounted to 87.4% of all participants, a total of 73,396 participants. Of the ELSA participants, the vast majority were White (96%), 3.9% were Non-White and 1.61% were of Asian ethnicity.

The Asian ELSA group were older than the population of LASI (63 years compared to 58 years), this may be in part due to the difference in minimum age for each study, with LASI allowing people aged 45 years or older instead of the 50 years plus criteria for ELSA. There were more females in the LASI cohort, compared to an almost equal split in the Asian ELSA. The Asian ELSA population had a higher BMI with almost half of them being overweight and 30% being obese. This difference in BMI was reflected in height and weight of each populous–the Asian ELSA being slightly taller but weighing 17–18 kg more. However this did not necessarily suggest that the Asian LASI group were overall healthier as over half of the LASI cohort were underweight. Further, this correlated to waist circumference where the LASI population had a much lower waist circumference compared to the Asian ELSA group. The Asian ELSA group had, on average, lower diastolic blood pressure but a higher systolic blood pressure, outside the normal range of blood pressure for both. Average resting heart rate was significantly higher in the LASI group (80 vs 55 bpm). The majority of LASI were educated to below secondary (64%) compared to 41% in Asian ELSA who were educated to below secondary. Moreover, three times more of the Asian ELSA group had obtained a degree or equivalent. More than half of LASI were unemployed, the opposite to Asian ELSA. Almost four-fifths of both groups were married.

Of the key physical performance variables included in the modelling analyses, the Asian ELSA generally performed better compared to the LASI group. Almost 79% of Asian ELSA achieved a maximum gait speed score of 4, compared to only 43% in the LASI group. Nobody in the LASI group achieved a standing balance score of 4, compared to almost half (49.7%) of the Asian ELSA achieving this. Grip strength, both dominant and non-dominant hand, were stronger in the Asian ELSA group compared to LASI, and time to complete the timed walk test was quicker, thus better, in the Asian ELSA compared to LASI.

Full details of baseline characteristics of all the different populations analysed in this paper are presented in Table 2. Physical performance variables were not normally distributed, thus median and interquartile range are presented for these variables.

**Table 2:** Baseline demographic characteristics and physical performance variables for LASI and ELSA subgroups.

		Total		Asian LASI		Asian ELSA		Non-white but not Asian ELSA		White ELSA		ELSA full sample	
	Number of participants	83,997		73,396		171		247		10,183		10,601	
**Baseline characteristics**	**Age (years)**	58.8	11.8	57.9	11.7	63.3	7.2	63.4	9.5	67.0	9.1	66.9	9.1
	**BMI (kgm** ^**-2**^ **)**	23.5	5.1	22.9	4.8	28.3	4.6	29.1	5.5	28.3	5.3	28.3	5.3
	**BMI group**												
	Underweight	36,805	49.9%	36,736	55.6%	1	1.0%	0	0.0%	68	0.9%	69	0.9%
	Normal	11,406	15.5%	9,363	14.2%	20	20.4%	36	25.5%	1,987	26.7%	2,043	26.6%
	Overweight	18,066	24.5%	14,892	22.5%	48	49.0%	49	34.8%	3,077	41.3%	3,174	41.3%
	Obese	7,519	10.2%	5,112	7.7%	29	29.6%	56	39.7%	2,322	31.2%	2,407	31.3%
	**Diastolic blood pressure (mmHg)**	80.9	0.1	81.7	10.4	76.9	10.7	74.8	10.8	73.5	10.8	73.6	10.8
	**Highest level of education**												
	None	2,655	5.3%	0	0.0%	52	31.1%	73	30.8%	2,530	25.2%	2,655	25.4%
	Foreign/other	1,204	2.4%	0	0.0%	13	7.8%	31	13.1%	1,160	11.6%	1,204	11.5%
	Below secondary	25,812	51.5%	25,382	64.0%	4	2.4%	12	5.1%	414	4.1%	430	4.1%
	Secondary or equivalent	8,781	17.5%	6,800	17.2%	18	10.8%	24	10.1%	1,939	19.3%	1,981	19.0%
	A-level or equivalent	5,960	11.9%	3,641	9.2%	32	19.2%	49	20.7%	2,238	22.3%	2,319	22.2%
	Degree or above	5,669	11.3%	3,811	9.6%	48	28.7%	48	20.3%	1,762	17.5%	1,858	17.8%
	**Employment status**												
	Not employed	13,121	53.0%	6,278	44.4%	98	57.3%	128	51.8%	6,617	65.0%	6,843	64.6%
	Employed	11,629	47.0%	7,871	55.6%	73	42.7%	119	48.2%	3,566	35.0%	3,758	35.5%
	**Heart rate (bpm)**	78.2	13.7	80.4	11.7	54.8	12.5	55.8	15.0	58.6	14.7	58.5	14.7
	**Height (cm)**	156.5	9.5	155.4	8.9	160.8	9.3	163.8	9.7	165.8	9.5	165.7	9.5
	**Marital status**												
	Married	63,298	75.4%	56,283	76.7%	143	83.6%	159	64.4	6,713	65.9%	7,015	66.2%
	Not married	20,695	24.6%	17,109	23.3%	28	16.4%	88	35.6	3,470	34.1%	3,586	33.8%
	**Sex**												
	Male	34,739	42.7%	31,135	42.4%	53	51.5%	68	46.6%	3,483	44.6%	3,604	44.8%
	Female	46,711	57.4%	42,261	57.6%	50	48.5%	78	53.4%	4,322	55.4%	4,450	55.3%
	**Systolic blood pressure (mmHg)**	128.6	19.1	128.2	19.2	131.8	15.4	130.6	16.7	132.1	17.5	132.0	17.5
	**Waist circumference (cm)**	86.7	13.4	85.6	12.9	96.5	12.0	98.1	12.6	96.4	14.0	96.4	13.9
	**Weight (kg)**	57.9	15.1	55.6	13.0	73.2	12.9	78.4	15.9	77.9	16.6	77.8	16.6
** Physical performance characteristics**	**Gait score**												
	1	2,696	3.8%	2,593	4.0%	4	6.1%	6	6.5%	93	1.5%	103	1.6%
	2	11,034	15.4%	10,868	16.6%	4	6.1%	5	5.4%	157	2.5%	166	2.6%
	3	24,013	33.5%	23,726	36.3%	6	9.1%	9	9.7%	272	4.3%	287	4.5%
	4	34,011	47.4%	28,124	43.1%	52	78.8%	73	78.5%	5,762	91.7%	5,887	91.4%
	Grip strength average (dominant) (kg)*	22	(17–28)	22	(17–28)	25.0	(18.3–34.7)	26.2	(20.7–35.0)	27	(21–36)	27	(21–36)
	Grip strength average (non-dominant) (kg)*	20	(15–26)	20	(15–25)	22.7	(14.7–30.3)	23.7	(19.0–33.3)	25	(19–33)	25	(19–33)
	**Standing balance score**												
	1	319	0.4%	319	0.4%	0	0.0%	0	0.0	0	0.0%	0	0.0%
	2	13,153	15.7%	9,964	13.6%	73	42.7%	111	44.9%	3,005	29.5%	3,189	30.1%
	3	63,971	76.2%	63,113	86.0%	13	7.6%	14	5.7%	831	8.2%	858	8.1%
	4	6,554	7.8%	0	0.0%	85	49.7%	122	49.4%	6,347	62.3%	6,554	61.8%
	Timed walk average (s)*	4.9	(4.2–5.8)	5.1	(4.3–5.9)	3.6	(2.8–4.6)	3.1	(2.6–4.2)	2.7	(2.3–3.4)	2.7	(2.3–3.4)

*median (inter-quartile range).

Abbreviations: BMI = body mass index; bpm = beats per minute; cm = centimetre; ELSA = English Longitudinal Study of Ageing; kg = kilograms; kgm^-2^ = kilograms per metre-squared;LASI = Longitudinal Ageing Study in India; mmHG = millimetres of mercury; NW = Non-white; s = seconds; W = White.

### 3.2. Primary analysis

The primary analysis compared cardiovascular disease in White ELSA to Non-White but not Asian ELSA, Asian ELSA, and Asian LASI, after adjusting for confounders. A categorical variable was created where the reference group was the White ELSA, and the other three ethnicities made up the other categories. This variable was forced into the model from the start of the modelling process.

A multivariate logistic regression model was modelled for the primary analysis to assess the odds of having CVD in the different ethnic groups, adjusting for the other confounders listed in the baseline characteristics section. Univariate analyses suggested every covariate was statistically significantly associated to CVD as p<0.10 for all. The final model consisted of ethnicity by way of race and which questionnaire participants partook in, age, diastolic blood pressure, systolic blood pressure, heart rate, sex, waist circumference, gait score, dominant handgrip strength, and standing balance score.

#### Factors associated with CVD across the full sample

Compared to the ELSA White ethnic group, LASI participants were significantly associated with a lower odds of CVD by 23% (95% CI = 0.60 to 0.99). When compared to the Asian group of ELSA, the LASI participants had a lower odds of CVD by around 40%, but this was not a statically significant result due to overlapping 95 Cis.

Higher age, systolic blood pressure and waist circumference were associated with higher odds of CVD. Conversely, higher diastolic blood pressure, resting heart rate, gait score, handgrip strength, balance score, and females were associated with lower odds of CVD. The constant term of 0.06 in the logistic regression model implies that, when all other covariates are held constant, the odds of CVD decreased are approximately 94% when compared to the baseline scenario where the predictor variables such as age and sex are at their reference values.

#### Comparison of CVD risk between Asian subgroups

Using the odds ratios from the model of the full sample, the odds ratio of Asian ELSA vs Asian LASI was calculated by dividing the respective odds ratios. This gives an OR of 1.65 (0.24, 11.50) for the Asian ELSA group against the reference group, Asian LASI. The Asian ELSA group have a 65% increased odds of CVD compared to the Asian LASI group, however this wide interval indicates a high degree of uncertainty in the estimate. The result is not statistically significant, possibly due to the difference in sample sizes between the two groups. The wide confidence interval suggests that the true odds ratio could vary widely, and the data do not provide sufficient evidence to conclude that there is a statistically significant difference in the odds of CVD between the Asian ELSA and Asian LASI groups.

Results of the primary analysis are presented in Table 3.

**Table 3:** Results of the multivariate logistic regression model of CVD.

	Primary analysis		
	OR	95% CI	
**Race and dataset**			
White ELSA (reference)	1.00		
Non-White but not Asian ELSA	0.99	0.39	2.57
Asian ELSA	1.27	0.44	3.69
Asian LASI	0.77	0.60	0.99
**Age (centred)**	1.03	1.03	1.04
**Diastolic blood pressure (centred)**	0.98	0.97	0.99
**Heart rate (centred)**	0.99	0.99	0.99
**Sex**			
Male (reference)	1.00		
Female	0.63	0.57	0.70
**Systolic blood pressure (centred)**	1.00	1.00	1.01
**Waist circumference (centred)**	1.04	1.03	1.04
**Gait score**			
1 (reference)	1.00		
2	0.95	0.79	1.15
3	0.98	0.81	1.19
4	0.77	0.63	0.94
**Dominant handgrip strength (centred)**	0.98	0.98	0.99
**Standing balance score**			
1 (reference)	1.00		
2	0.84	0.52	1.37
3	0.86	0.55	1.36
4	0.45	0.27	0.76
**Constant**	0.06	0.04	0.11

#### 3.2.1 Testing assumptions

The outcome of the specification error test concluded that both meaningful predictors were chosen for the model, $p(\hat{h})$ = 0.001, and that no relevant variables were omitted $p(\hat{h^{2}})$ = 0.152. The pseudo R^2^ of the model was 0.0655, indicating that around 6.6% of the variable in the model can be explained by the variables included in the model. The Hosmer and Lemeshow’s goodness-of-fit test concluded good fit (p = 0.3633).

Multicollinearity was tested for the continuous covariates included in the final model: age, diastolic blood pressure, resting heart rate, and waist circumference. VIF was between 1.09–1.98 and tolerance was between 0.82–0.97 for all variables, indicating that the variables were orthogonal (unrelated) to each other, thus multicollinearity was not a factor in the model.

When re-running the primary analysis model without observations with high values of studentised Pearson residuals, deviance residuals, or highly influential observations, there were no statistically significant differences in estimates. Furthermore, only the model where deviance residuals >|2| were removed were the estimates different to the final model. Despite this, statistical significance was remained unchanged.

#### 3.2.2 Sensitivity analysis

Table 4 presents the results of the three sensitivity analyses conducted and how they compare to the results of the multivariate model presented in section 3.2. Primary analysis.

**Table 4:** Results of sensitivity analyses on ethnic groups.

	Primary analysis			Sensitivity analysis 1			Sensitivity analysis 2				Sensitivity analysis 3			
Race and dataset	OR	95% CI		OR	95% CI		OR		95% CI		OR		95% CI	
White ELSA (reference)	1.00			1.00			1.00				1.00			
Non-White but not Asian ELSA	0.99	0.39	2.57	0.99	0.33	2.94	0.69	0.64		0.75	0.52	0.52		0.53
Asian ELSA	1.27	0.44	3.69	1.24	0.36	4.21	1.05	0.98		1.13	0.91	0.90		0.92
Asian LASI	0.77	0.60	0.99	0.78	0.61	0.99	1.07	0.95		1.20	0.54	0.54		0.55

The first sensitivity analysis randomly sampled one-third of the Asian ELSA group under the assumption of these participants being of Indian ethnicity. This was done 1,000 times. The results of sensitivity analysis 1 was consistent with the primary analysis. The odds of CVD remained highest for the Asian ELSA group, but this result was not statistically significant.

The second sensitivity analysis used bootstrapping to inflate the sample sizes of White ELSA, Non-White but not Asian ELSA, and Asian ELSA to be the same as Asian LASI. Again, this sampling method was repeated 1,000 times. Compared to White ELSA, both Asian groups were associated with slightly, but not significant, higher odds of CVD. Contrary to the primary results, Asian LASI exhibited higher, but not statistically significant, odds of CVD compared to Asian ELSA by 2%. 2%. The Non-White but not Asian ELSA group were associated with the lowest odds of CVD, a statistically significant decrease of 31% compared to White ELSA.

The third sensitivity analysis used bootstrapping to deflate the sample sizes of White ELSA, Non-White but not Asian ELSA, and Asian LASI to be the same as Asian ELSA. The model was performed 1,000 times. All three other ethnicities were associated with statistically significant lower odds of CVD compared to White ELSA. Moreover, Asian LASI was associated with 41% lower odds of CVD compared to Asian ELSA.

Across all three sensitivity analyses, the Non-White but not Asian ELSA group consistently performed the best. The Asian LASI group was showed significantly lower odds of CVD compared to White ELSA, except in sensitivity analysis 2. In sensitivity analysis 3, Asian ELSA exhibited a statistically significant lower odds of CVD compared to White ELSA, consistent with the primary analysis. Conversely, Asian LASI displayed a significantly lower odds ratio in sensitivity analysis 3 compared to White ELSA, contrasting the primary analysis where no significant difference was observed. Furthermore, the comparison between Asian ELSA and Asian LASI demonstrated that Asian ELSA had a significantly higher odds ratio in sensitivity analysis 3. These findings emphasize the importance of considering sensitivity analyses to uncover nuanced associations, underscoring the potential impact of dataset variations on outcomes within Asian populations, and how the differing ample sizes of each group plays a part in the result found.

## 4. Discussion

### 4.1. General discussion

This study investigated factors that predicted the odds of CVD for four different populations, Whites, Asians, and non-Asians in the UK and Asians in India. The present study found that there was no statistically significant difference between the Asian LASI and Asian ELSA group, only a numeric difference where the Asian LASI group had lower CVD odds in comparison, prompting consideration of potential trends that warrant further investigation. In contrast, there was a statistically significant difference between the Asian LASI and White ELSA groups, where the Asian LASI group were associated with lower odds of CVD. This observation underscores potential ethnic disparities in CVD risk factors which demand closer scrutiny. Our study reveals notable differences in cardiovascular disease (CVD) risks among the UK Asian diaspora and residents of India. Specifically, the Asian ELSA subgroup, characterized by an older age profile and higher BMI, exhibited different risk factors compared to the LASI participants. This suggests a significant influence of environmental and lifestyle factors in the diaspora. The lower odds of CVD in LASI participants compared to the White ELSA group underline the potential impact of ethnic and geographic differences on health outcomes.

The present study highlights significant differences in CVD risks among the UK Asian diaspora and LASI participants, with notable variations in baseline blood pressure, heart rate, and anthropometric measures. While our analysis revealed consistent associations between traditional CVD risk factors and disease outcomes across different ethnic groups, unexpected findings, such as the inverse relationship between diastolic blood pressure and CVD odds, warrant further investigation.

Our analysis indicates that higher age, systolic blood pressure, and waist circumference are crucial risk factors for CVD, consistent across different ethnic groups. This underscores the importance of these indicators in public health screening and intervention strategies. On the other hand, factors like higher resting heart rate, physical fitness (as indicated by gait score and handgrip strength), and being female are associated with reduced CVD risk, pointing towards the protective role of physical fitness and gender-specific health patterns. However, unexpected results also emerged, like elevated diastolic blood pressure correlating to reduced CVD odds, contradicting most research tying higher diastolic levels to amplified risk [37–39]. This trend remained consistent across both primary and sensitivity analyses, indicating an underlying predictor variable or sample characteristic driving the anomaly.

The sensitivity analyses provide a deeper understanding, revealing how variations in sample size and ethnic composition can influence CVD risk assessments. The consistent lower odds of CVD in the Non-White but not Asian ELSA group across different analyses suggest that ethnic and cultural factors play a significant role in health outcomes. The varying risk profiles between Asian ELSA and Asian LASI participants further emphasize the complexity of CVD risk factors, influenced by a blend of genetic, environmental, and lifestyle factors.

This study aligns with existing literature which have previously shown differences in CVD risk and outcomes between ethnicities in developed countries [40,41]. In the UK, South Asian women having significantly lower odds of CVD compared to the general population [42]. However, no studies were found that compared the localised ethnicity, in this case Asians, versus the diaspora of that ethnicity.

Traditional risk factors such as age, blood pressure, resting heart rate, sex, waist circumference, handgrip strength, gait and balance were associated with CVD. These associations were consistent with established risk factors in previous research. However, the unexpected decrease in CVD odds with increasing diastolic blood pressure prompts a call for further investigation.

Age was associated to a statistically significant 3% increase in CVD odds throughout primary and sensitivity analyses, corresponding to past research stating age as a key factor in CVD risk [43]. Gender was significantly associated to CVD, namely that women had 37% decreased odds of CVD. This result also corresponds to previous data and may be, in part, due to biological factors such as genes and hormones [44,45] but this link is still not properly understood. Moreover, CVD is still under-recognised and untreated in women [46]. An in-depth exploration of gender differences and the potential impact of biological factors on CVD risk in women would enhance the discussion.

Blood pressure is associated with strong evidence for causation of CVD [47]. The present study found that an increase in diastolic blood pressure is associated with a 2% decrease in CVD, but increases in systolic blood pressure increased odds of CVD under 1%. A study from seven diverse US cohorts studies found that increases in blood pressure above normal levels were associated with increases in lifetime risk of CVD [48,49] which contradicts the findings in this paper regarding diastolic blood pressure.

Increases in resting heart rate compared to the average of 60 BPM was associated with a 1% reduction in CVD odds throughout the analyses. The results of the present study were in the opposite direction to results in another study [50] where low heart rate reduced risk for CVD. Another study used reference groups of 60 bpm for males and 64 bpm in females [51]. Per a 15-bpm increase, risk of CVD significantly increased by 24% in men and 32% in women. This discrepancy in the magnitude and direction of the association prompts further investigation into potential factors contributing to these disparities, including differences in study populations, methodologies, or covariates considered.

One centimetre increases in waist circumference was associated with a 4% increase in CVD odds, echoing other studies where waist circumference is strongly correlated with CVD risk [52]. High waist circumference is an indicator for diseases such a type II diabetes, hypocholesteraemia, joint pain, and other non-communicable disease [53].

Physical performance variables were significantly associated to CVD in the primary analysis, after adjusting for confounders. Dominant handgrip strength was associated with a 2% lower CVD risk, higher gait score was associated with up to 23% decreased odds of CVD for the fastest gait speed, and standing balance was associated with up to a 55% decrease in CVD odds for the group with the highest standing balance score. Consideration of potential confounding variables related to central obesity could further enrich this discussion.

A longitudinal study of 962 South Indians found that CVD risk factors, such as anthropometric and biological measures, was more prevalent among Indians compared with high and upper middle income countries [54], this may be explained in part by findings of another study [55] where patients of SA origin, based in India and the UK, had limited knowledge about CVD risk.

Results from post-hoc analyses of the PURE study found that grip strength was a significant predictor of CVD, a stronger predictor than that of physical activity, but a weaker predictor compared to systolic blood pressure [56] which was opposite to the findings of this study albeit still statistically significant. Another paper analysing UK Biobank data [18] concluded that including grip strength and walking pace improved CVD risk prediction. Gait and balance disorders are a major cause of falls in older adults and associated with increased morbidity and mortality [57–59].

There were both consistencies and disparities between the results presented in this paper and other published research which underscores the important of exploring these factors further, and the need for comprehensive, context-specific analyses in CVD and ethnicity given the paucity of evidence comparing ethnic diasporas with their local counterparts. Our findings suggest the importance of considering ethnic and cultural factors in cardiovascular risk assessment and intervention strategies, highlighting the need for targeted approaches tailored to the unique needs of different ethnic groups.

### 4.2. Strengths and limitations

To the best of the authors’ knowledge, this is the first paper that directly compared Asians in the UK to those in India, using two sister-studies which used many of the same questionnaires and measured the same outcomes, increasing the comparability between a study conducted in the UK to a study conducted in India.

The observed differences in baseline characteristics, namely BMI, diastolic blood pressure, heart rate, waist circumference, and weight, between the LASI and ELSA participants may introduce confounding factors that could influence CVD-related outcomes. While these differences offer valuable insights into potential risk factors, factors such as genetic predispositions, cultural practices, dietary habits, access to healthcare, and socioeconomic status could all contribute to the observed differences, complicating the interpretation of study findings. While these baseline characteristics offer important avenues for further investigation, caution must be exercised in attributing causality solely to these factors.

There was a 5-year difference in the waves analysed between ELSA and LASI, however this inconsistency was justified as it allowed direct comparison between the comparable waves of either study and was not a large inconsistency between timepoints.

The large difference in sample sizes between the Asian population in ELSA and the overall LASI population is a limitation of this study. Although the small number of the Asian and British Asian population in ELSA is to be expected, it decreases the precision and reliability of results compared to if the sample size was adequate. Similarly for the Non-White cohort of ELSA which had a relatively small sample size.

Alcohol consumption use is a known risk factor of CVD but was not included in the analysis as laws regarding the sale and consumption of alcohol in India vary from state to state. Alcohol is totally illegal in five states (Bihar, Gujarat, Lakshadweep, Mizoram, and Nagaland). Some states such as Kerela and Tamil Nadu allow only the state government to sell alcohol, not independent retailers, and others such as Delhi prohibit the home delivery of alcoholic beverages. Due to varying state laws on alcohol sales in India, and unequal access to alcohol, it was excluded as a risk factor in the analysis.

Statistical analyses were planned prior to accessing the data which was comprehensive, all major assumptions were tested regarding the model used in the analysis, as well as exhaustive post-estimation and sensitivity analyses which were likewise thorough.

### 4.3. Implications and future research

The findings in this study have shown that Asians living in India had a lower risk of CVD compared to Whites living in England in the 21^st^ century. This may be explained by factors exclusive to England compared to India, such as differences in diet, lifestyle, social life, exercise, among others. Moreover, this study found men were significantly associated to CVD compared to women, and the importance of physical strength, blood pressure, and waist circumferences, as proxies of health, in combating the development of CVD.

Differences in environmental factors and societal norms between India and England may play a crucial role in understanding the observed variations in CVD risk. For instance, dietary patterns rich in specific nutrients, variations in daily physical activity due to occupational or lifestyle differences, and cultural practices that influence overall well-being may contribute to the divergent CVD outcomes. Investigating these contextual factors can provide a more comprehensive understanding of the nuanced influences on cardiovascular health.

Understanding the impact of lifestyle factors, gender, and physical health indicators on CVD risk has practical implications for preventive strategies and interventions. Tailoring public health initiatives to address specific risk factors, such as promoting healthier lifestyle choices, encouraging regular physical activity, and emphasizing the importance of maintaining a healthy weight, may contribute to reducing CVD incidence and improving overall cardiovascular health.

Thus far, there is only one wave of LASI data available to analyse. In the future, more waves will be available, allowing researchers to investigate and identify developmental trends in the various categories of data collected in LASI. Although steps were taken to ensure comparability between the waves in terms of when the data were collected between the two studies, it should be noted that the results presented may only be applicable to this time period as analysing one time point restricts the ability to understand how variables evolve or interact over the course of the study and ignores individual variability.

Moreover, death data will become available for LASI participants, which will allow researchers to investigate the factors related to all-cause and cause-specific mortality. This is especially useful if the comparison to Asians, and other ethnicities, in ELSA, or other longitudinal datasets, is being made. Data on other ethnicities will become available through the other sister-studies of the HRS, allowing the comparison between mortality between other localised and diaspora ethnicities, Factors such as gender and employment should be explored in more detail to ascertain plausible reasons as to why they are significantly associated with CVD.

Finally, more data should be collected on Asians and other ethnic minorities when conducting studies in the UK. Despite ELSA being largely representative, sample sizes of the minority ethnics were far too low. Comparable sample sizes of these ethnicities would allow researchers to see whether the notion of increased risk in Asians, for example, in the UK is accurate.

### 4.4. Conclusions

To conclude, the present study investigated how CVD differed between ethnic groups in the UK to Asians from India and the factors associated to CVD. After adjusting for risk factors, there was a statistically significant difference in CVD odds between Whites in the UK to Indians, with Indians at a lower risk. Asians in the UK were not statistically difference to Asians in India in terms of CVD risk, however a limitation is the small number of ethnic minorities included in ELSA. Further longitudinal work, which will be available in the coming years, allow investigation of mortality. Additionally, future studies with comparable sample sizes and more potential risk factors measured will be useful in understanding why disparities between these different populations arise with respect to CVD. Overall, our findings contribute to a more nuanced understanding of CVD risks, highlighting the need for tailored public health strategies that consider ethnic diversity, lifestyle factors, and socio-economic status. This study not only adds to the existing literature on CVD but also underscores the importance of comprehensive and culturally sensitive healthcare approaches.
